# Biomarkers reflecting insulin resistance increase the risk of aortic stenosis in a population-based study of 10,144 Finnish men

**DOI:** 10.1080/07853890.2024.2419996

**Published:** 2024-11-26

**Authors:** Maija Sohlman, Raimo Jauhiainen, Jagadish Vangipurapu, Annamaria Laakso, Mika Ala-Korpela, Teemu Kuulasmaa, Johanna Kuusisto

**Affiliations:** a Institute of Clinical Medicine, Internal Medicine, University of Eastern Finland, Kuopio, Finland; b Center for Medicine and Clinical Research, Kuopio University Hospital, Kuopio, Finland; c Systems Epidemiology, University of Oulu, Oulu, Finland; d Research Unit of Population Health, University of Oulu, Oulu, Finland; e Biocenter Oulu, University of Oulu, Oulu, Finland; f NMR Metabolomics Laboratory, School of Pharmacy, University of Eastern Finland, Kuopio, Finland

**Keywords:** Aortic stenosis, insulin resistance, insulin, biomarkers, principal component analysis

## Abstract

**Aims:**

To investigate a comprehensive panel of biomarkers and risk of aortic stenosis (AS) in a prospective population-based study.

**Methods:**

Anthropometric, metabolic, and inflammatory biomarkers were measured in the Metabolic Syndrome in the Men Study of 10,144 Finnish men without AS at baseline. Cases of AS were identified from the medical records. Cox regression analysis was used to identify variables predicting AS over a follow-up time of 10.8 years. Principal component (PC) analysis was applied to the biomarkers that predicted AS. Cox regression analysis was used to investigate the resulting PCs as AS predictors.

**Results:**

AS was diagnosed in 116 men (1.1%), with a median age of 62 years. In Cox regression analyses, fasting, 30 min, and 120 min plasma insulin, and proinsulin, with hazard ratios (HR) ranging from 1.38 (1.12-1.69, *p* = 2.1E-3) to 1.44 (1.23-1.68, *p* = 4.0E-6), Matsuda index [HR 0.68 (0.56-0.82, *p* = 6.9E-5)], and serum C-peptide [HR 1.47 (1.22-1.77, *p* = 5.0E-5)] were associated with incident AS, in addition to age, systolic blood pressure, BMI, waist circumference, waist/hip ratio, height, body fat mass, fat-free mass, and hs-CRP, and remained significant after adjustments, or if diabetic subjects were excluded. PC 1, consisting of fasting plasma insulin, C-peptide, Matsuda index, waist/hip ratio, and urine albumin excretion, and PC 2, consisting of age, body fat mass, and systolic blood pressure, were significantly associated with AS [HRs 1.37(1.09-1.73) and 1.77 (1.45-2.17), respectively].

**Conclusion:**

Biomarkers reflecting insulin resistance are risk factors for AS, a novel finding indicating that insulin resistance is important in the pathogenesis of AS.

## LAY SUMMARY

Aortic stenosis is an active atherosclerosis-like process that leads to progressive aortic valve calcification and obstruction. The present study showed that insulin resistance and hyperinsulinemia predict aortic stenosis independent of other cardiovascular risk factors, such as diabetes, obesity, and systolic blood pressure, suggesting that insulin resistance is an important factor in the pathogenesis of aortic stenosis.

## KEY MESSAGES

- In the present large-scale population-based follow-up study, several biomarkers reflecting hyperinsulinemia and/or insulin resistance, including fasting, 30 minutes, and 120 minutes plasma insulin and proinsulin, Matsuda index, and serum C-peptide, were associated with incident AS, which is to the best of our knowledge a novel finding.
- Biomarkers of insulin resistance associated with incident AS, even after adjustments for BMI and hypertension, or exclusion of diabetic subjects or those with a nontricuspid aortic valve.

## Introduction

Aortic stenosis (AS) is the most prevalent valvular heart disease in western countries. The prevalence of AS increases from 0.1% in those aged 50 years to 2.6% in subjects aged 75 years or over [1]. In 5 years, if untreated, severe AS has 70-80% risk of death [2]. To date, aortic valve replacement by open-heart surgery or, more recently, transcatheter aortic valve replacement (TAVR) has been the only treatment for AS.

Previously, AS was considered degenerative; however, in the nineties, it was identified as an active inflammatory process similar to atherosclerotic vascular disease [3]. Progressive thickening and fibrocalcific remodeling of the aortic valve leaflets result from endothelial damage, accumulation of plasma lipoproteins, chronic inflammatory infiltrate, extracellular matrix proteins, and active calcification [3, 4]. In calcific aortic valve disease (CAVD), valve lesions are present in early adulthood but increase in size and progress with calcification in middle-aged and elderly individuals, eventually leading to valve obstruction and AS [3, 5].

The bicuspid aortic valve, a congenital malformation present in 1% of the population [6], is the most important risk factor for AS. Almost half of aortic valve replacements in AS are performed in patients with a bicuspid aortic valve. In cross-sectional studies, conventional cardiovascular risk factors including age, male sex, elevated low-density lipoprotein (LDL) cholesterol, lipoprotein(a), hypertension, smoking, and diabetes have been found to be associated with AS [1–7]. There are, however, only a few follow-up studies have investigated predictors of AS. In these studies, age, hypertension, body mass index (BMI), diabetes, dyslipidemia, high CRP, smoking, LDL cholesterol, and Lp (a) levels [8–13] were associated with incident AS. In 5.4 million patients in the UK, systolic blood pressure was continuously related to the risk of AS [^9^]. In large-scale genetic studies, NOTCH1 mutations [14], genetically determined Lp (a) levels [13], LDL cholesterol genetic risk scores [12], genetically associated increments in systolic blood pressure [15], and genetically increased body mass index [16] are causally related to AS.

Clinical trials have failed to demonstrate a significant effect of statins on slowing the progression of AS [17], suggesting that LDL cholesterol may not be as important in the pathogenesis of AS as it is in coronary artery disease. Recent genetic studies have shown that the etiology of CAVD is multifactorial. Presumably, several factors interact with the initiation and progression of the disease in the pathogenesis of AS. To find a means for early prevention of AS, it is crucial to identify how different factors contribute to the disease process. However, there have been no studies on large panels of biomarkers as risk factors for AS. Therefore, we investigated which factors of a comprehensive panel of biomarkers, including biomarkers of insulin resistance, inflammation, and metabolites derived from proton NMR analysis, were associated with the development of AS in a population-based study of 10,144 Finnish men without AS at baseline.

## Methods

### Subjects and measurements at baseline

The Metabolic Syndrome in Men (METSIM) Study included 10,197 men, aged from 45 to 73 years at baseline, randomly selected from the population register of Kuopio, Eastern Finland (population of 95,000 at the time of the baseline study). At baseline, every subject participating in the study had a 1-day outpatient visit to the Clinical Research Unit at the University of Eastern Finland [18], where anthropometric measurements and blood samples for metabolic, inflammatory, proton nuclear magnetic resonance (NMR) analysis, and other biomarkers were taken. The design and methods of the METSIM Study have been previously described [18] For detailed methodology of anthropometric measurements, oral glucose tolerance test, Matsuda index, C-peptide, diagnosis of diabetes, laboratory measurements including inflammatory biomarkers, and the protocol of proton nuclear magnetic resonance analysis see supplementary data. In the present study, all participants with prevalent AS before the baseline study (*n* = 53) were excluded from the statistical analyses, resulting in a final study population of 10,144 men. Baseline measurements were available for all 10,144 subjects (Tables S2 – S5).

### Diagnosis of AS

All AS diagnoses (International Classification of Diseases, 10th Revision [ICD-10]: I 35.0, I35.2) were obtained from the patient registries of the Kuopio University Hospital and local Harjula Hospital of Kuopio, which are the only hospitals with cardiology outpatient clinics that diagnose, follow, and treat subjects with AS in the living area of the METSIM study subjects. Hospital registries were reviewed regularly and comprehensively until the end of the follow-up of the present study. Diagnoses of AS were based on clinical diagnoses of AS made by cardiologists at the Kuopio University Hospital and the Harjula Hospital. The diagnoses and clinical and echocardiographic findings were confirmed from individual medical charts. One of the study researchers (M.S.) reviewed the medical charts of all METSIM study subjects with a diagnosis of AS made before and after baseline. Clinically, the subjects with AS were followed in the cardiology clinic according to the international guidelines. Data collection from the medical charts was supervised by a cardiologist with a 23-year experience in clinical cardiology, echocardiography, and research on aortic stenosis (J.K.) Detailed data of symptoms related to AS, ECG, and echocardiographic parameters at the time of diagnosis were collected. The diagnosis of AS was defined as CAVD with a maximal aortic valve gradient ≥20 mmHg on transthoracic echocardiography performed by a cardiologist. Echocardiographic assessments of AS followed the international recommendations [19, 20].

### Study follow-up and time to diagnosis of incident aortic stenosis

The study participants were followed-up for development of AS until 25 September 2018, for a mean period of 10.8 ± 1.4 years (median 10.8 years, range 8.3 − 13.5 years). Mean time from the baseline examination to the diagnosis of incident AS was 6.4 ± 3.1 years (median 6.8 years, range 0.5 − 12.3 years).

### Statistical analysis

Statistical analyses were performed using SPSS version 23 (SPSS Inc., Chicago, IL, USA). Data are presented as the mean ± SD, or median, when appropriate. All variables, except age, were log-transformed to correct for skewed distribution. Independent samples *t-*test and Pearson χ2 test were used to assess the statistical significance of the differences in anthropometric and laboratory biomarkers and proton NMR metabolic measures between participants with and without incident AS. Cox regression analysis and hazard ratios (HR) were used to investigate the association between baseline variables and incident AS. The anthropometric parameters and biomarkers, which differed significantly in the independent samples *t*-test between the subjects with and without incident AS, were included in the Cox regression analyses, except for total cholesterol and LDL cholesterol, which were excluded from the analyses due to possible bias caused by high statin use in subjects with AS. A value **<** 0.05, divided by the number of comparisons was considered statistically significant. Principal component (PC) analysis, a statistical procedure that converts a set of observations of correlated variables into a set of values of linearly uncorrelated variables called PCs, was applied to analyze the baseline variables associated with incident AS in the unadjusted Cox regression analysis. Associations between the resulting PCs and incident AS were investigated using Cox regression analysis.

### Ethics statement

The METSIM study protocol was approved by the Ethics Committee of Kuopio University Hospital (171/2004). This study was conducted in accordance with the principles of the Declaration of Helsinki. All the study participants provided written informed consent and agreed to follow-up by the registries without time limit. The authors have permission from the registry owners, Kuopio University Hospital and Harjula Hospital, to access the data used in this study.

## Results

Incident AS was diagnosed in 116 (1.1%) men in the METSIM Study over a mean follow-up time of 10.8 years. In subjects with incident AS, the median age at baseline METSIM examination was 62 years (range: 45–73 years). Table S1 presents the clinical characteristics, symptoms related to AS, and ECG and echocardiographic findings of subjects with incident AS at the time of AS diagnosis. On echocardiography, the maximal aortic valve gradient was 35.5 ± 18.4 mmHg. Most patients had tricuspid aortic valves. Bicuspid aortic valve was found in about one-fifth of the subjects, and in another fifth of the subjects, the number of cusps was impossible to define or was not mentioned in the medical records. Concomitant mild aortic valve regurgitation was present in approximately 50% of patients.

Table S2 presents the METSIM baseline characteristics of the study participants with and without incident AS. Age, systolic blood pressure, body mass index, waist circumference, waist-hip ratio, and body fat mass percentage were significantly higher and fat-free mass percentage was lower in those who developed AS than in those who did not develop AS during follow-up. Diabetes and heart failure were significantly more common at baseline in patients with incident AS. Subjects with incident AS had nominally significantly shorter height, decreased body muscle mass, and more peripheral artery revascularization and carotid artery operations, but there was no difference in the prevalence of previous coronary artery revascularization between those with or without incident AS. The use of metformin and statins was significantly more frequent in the subjects with incident AS (data not shown).

**Figure 1.**
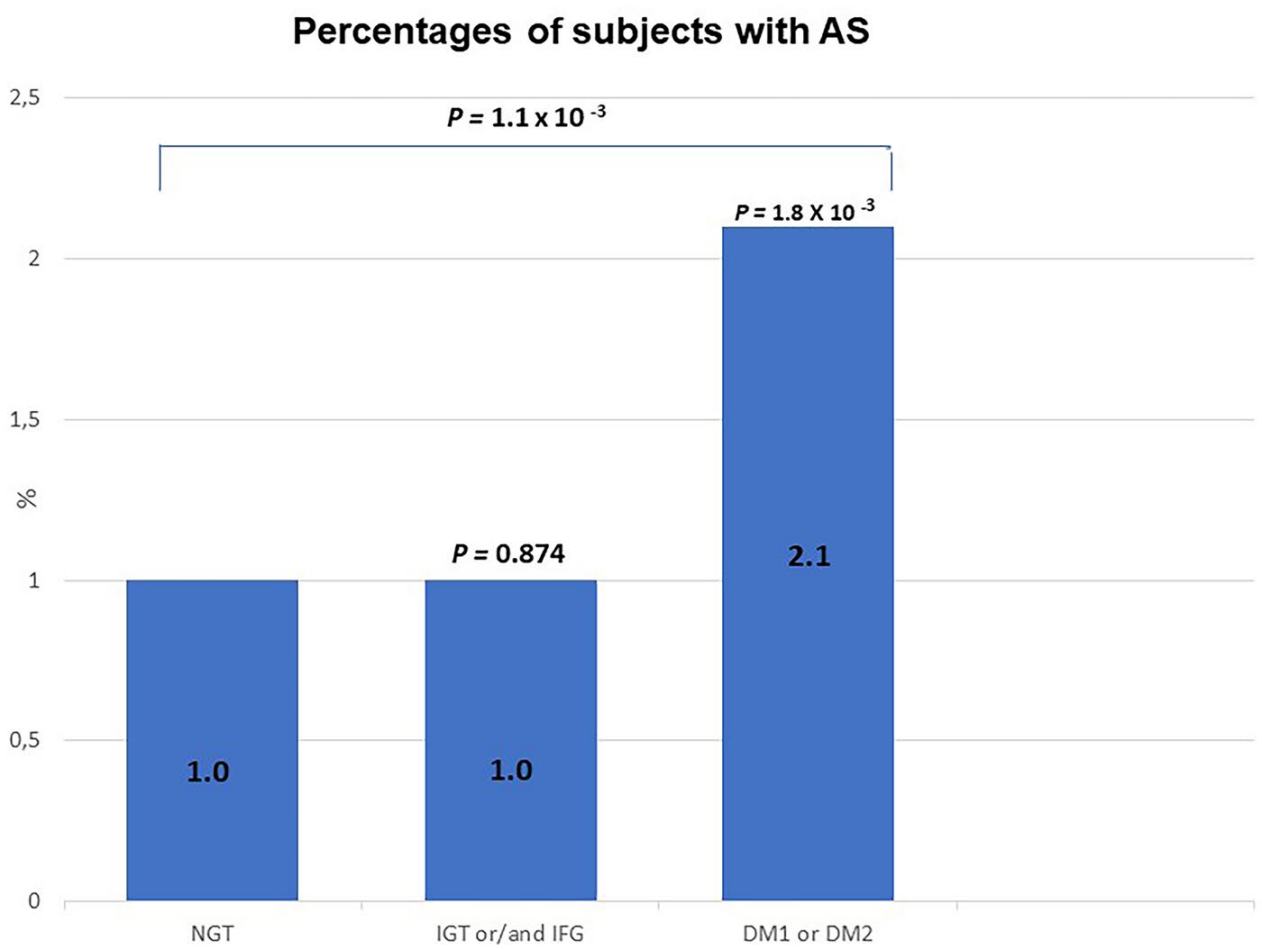
Percentages of subjects with incident AS in three glucose tolerance groups. NGT: normal glucose tolerance; IGT: impaired glucose tolerance; IFG: impaired fasting glucose. Analyses include 10,144 study participants. Individuals with AS at baseline (*n* = 53) are excluded. *P*: Pearson Chi-square test over all three groups *p*: Pearson’s chi-square test over 1. normal vs. IFG and/or IGT, and 2. Normal vs. diabetic glucose tolerance group.

Figure 1 shows the percentage of participants with incident AS in each glucose tolerance group. There was a significantly higher incidence of AS in subjects with diabetes than in those with normal glucose tolerance (NGT), but there was no significant difference in the incidence of AS between those with NGT and impaired fasting glucose or impaired glucose tolerance.

Table S3 shows the baseline metabolic and inflammatory biomarkers in subjects with and without incident AS. Subjects with incident AS had higher urine albumin, urine albumin excretion rate, and hs-CRP levels than those without incident AS. The total and LDL cholesterol concentrations were nominally lower in patients with incident AS than in those without incident AS. There were no significant differences between the groups in the levels of total triglycerides, ApoA1, ApoB, alanine transaminase, creatinine, eGFR Modification of Diet in Renal Disease (MDRD), eGFR Cockroft-Gault, adiponectin, interleukin**-**1 receptor antagonist, interleukin-1 beta, or glycoprotein acetyls.

Table S4 presents the biomarkers of glucose and insulin metabolism in the subjects with and without incident AS. Subjects with incident AS had higher levels of fasting plasma insulin and proinsulin, OGTT 30 min plasma insulin and proinsulin, OGTT 120 min plasma insulin and proinsulin, and serum C-peptide levels than those without AS. The Matsuda index was significantly lower in subjects with AS. Fasting plasma glucose and HbA1c levels were nominally higher in patients with incident AS than in those without AS.

Table S5 shows amino acids, fatty acids, lipids, and ketone bodies measured by proton NMR analysis in study participants with and without incident AS. Nominally significant differences between groups were found in histidine, phenylalanine, fasting plasma free fatty acids, total phosphoglyserides, apolipoprotein A1, and 3-hydroxybutyrate levels.

Tables 1 and 2 present the univariate Cox regression analyses of the associations between baseline anthropometric factors and biomarkers and incident AS. Variables that differed between the subjects with and without incident AS in the independent sample *t*-test with statistically significant p-values (Tables S2-S4), and fasting plasma glucose, GHbA1c, and body muscle mass percentage, which differed nominally between the study groups, were included in the Cox regression analyses. In unadjusted Cox regression analyses, all anthropometric and biomarkers included were associated with higher risk of AS with statistically significant hazard ratios ranging from 1.24 to 1.85 (from 0.63 to 0.80 with negatively associated biomarkers), except for fasting plasma glucose and blood HbA1C, which had nominally significant associations with AS. When adjusted for age, the factors significantly or nominally associated with incident AS were systolic blood pressure, body mass index, waist, waist/hip ratio, body fat mass and fat-free mass percentages, urine albumin, urine albumin excretion rate, hs-CRP, fasting plasma insulin and proinsulin, OGTT 30 min plasma insulin and proinsulin, OGTT 120 min plasma insulin and proinsulin, serum C-peptide and Matsuda index. Finally, when adjusted for age, BMI, smoking status, and reimbursement for hypertension and diabetes, the following biomarkers were nominally associated with incident AS: systolic blood pressure, fasting plasma insulin and proinsulin, OGTT 30 min plasma proinsulin, and serum C-peptide.

**Table 1.** Cox regression analysis of anthropometric variables as predictors for incident AS in 10,144 participants of the 10.8-year follow-up study of the METSIM cohort.

	N NON-CASE/CASE	HR	95 %Cl		*P*	P*	P**	P***	P****
			Lower	Upper					
Conventional risk factors									
Age	10,028/116	1.85	1.53	2.24	**4.0E-10**				
Systolic blood pressure	10,027/116	1.54	1.30	1.83	**7.3E-7**	**4.8E-4**	**4.3E-3**	**4.8E-3**	6.2E-3
Body mass index	10,025/115	1.31	1.10	1.54	**1.9E-3**	**1.7E-3**			
Waist	10,023/115	1.33	1.12	1.58	**1.1E-3**	**2.9E-3**	0.725	0.808	0.910
Waist/hip ratio	10,022/115	1.38	1.15	1.64	**4.5E-4**	**3.7E-3**	0.262	0.325	0.395
Height	10,025/115	0.77	0.64	0.92	**4.0E-3**	0.201	0.243	0.257	0.253
Bioimpedance									
Body fat mass percentage	10,005/114	1.74	1.42	2.12	**6.5E-8**	0.017	0.628	0.665	0.648
Body muscle mass percentage	10,005/114	0.80	0.68	0.93	**4.3E-3**	0.811	0.251	0.225	0.194
Body fat-free mass percentage	10,005/114	0.63	0.54	0.73	**3.0E-9**	7.6E-3	0.461	0.494	0.488

The analysis included 10,144 participants without incident AS at baseline, of whom 116 participants developed incident AS during the follow-up period. Participants with AS at baseline (*n* = 53) were excluded from the study. The hazard ratios (HR) were standardized. *p* < 5.6 x 10^−3^ (0.05/9) is considered statistically significant (bold). *p* < 0.05 is considered as nominally significant (underlined). P unadjusted. P* adjusted for age. P** adjusted for age and BMI. P*** adjusted for age, BMI, smoking status, and reimbursement for hypertension. P**** adjusted for age, BMI, smoking status, and reimbursement for hypertension and diabetes.

CI: confidence interval.

**Table 2.** Cox regression analysis of metabolic biomarkers as predictors for incident AS in 10,144 participants of an 10.8-year follow-up study of the METSIM cohort.

	N NON-CASE/CASE	HR	(95 %Cl)		*P*	P*	P**	P***	P****
			Lower	Upper					
Urine albumin	9,888/116	1.24	1.09	1.42	**1.6E-3**	0.049	0.376	0.466	0.682
Urine albumin excretion rate	9,882/116	1.27	1.11	1.46	**7.3E-4**	7.3E-3	0.136	0.165	0.286
High sensitive CRP	10,026/116	1.31	1.10	1.56	**2.4E-3**	4.5E-3	0.058	0.076	0.079
Fasting plasma glucose	10,028/116	1.18	1.02	1.36	0.023	0.095	0.591	0.650	0.716
Blood HbA1c	10,005/116	1.22	1.06	1.40	4.6E-3	0.139	0.766	0.862	0.597
Fasting plasma insulin	10,024/116	1.44	1.23	1.68	**4.0E-6**	**2.0E-5**	8.3E-3	0.011	0.026
OGTT 30 min plasma insulin	9,259/95	1.43	1.17	1.74	**4.4E-4**	**5.2E-4**	0.022	0.028	0.031
OGTT 120 min plasma insulin	9,295/95	1.38	1.12	1.69	**2.1E-3**	0.023	0.339	0.392	0.350
Fasting plasma proinsulin	10,026/116	1.40	1.20	1.65	**3.0E-5**	**1.5E-4**	0.034	0.043	0.098
OGTT 30 min plasma proinsulin	9,270/95	1.44	1.18	1.76	**3.6E-4**	**3.3E-4**	0.017	0.025	0.023
OGTT 120 min plasma proinsulin	9,300/95	1.39	1.13	1.71	**1.6E-3**	0.021	0.173	0.217	0.191
Serum C-peptide	3,267/47	1.47	1.22	1.77	**5.0E-5**	**4.9E-4**	0.028	0.038	0.049
Matsuda index	9,248/95	0.68	0.56	0.82	**6.9E-5**	**2.9E-4**	0.049	0.079	0.055

The analysis included 10,144 participants without incident AS at baseline, of whom 116 participants developed incident AS during the follow-up period. Participants with AS at baseline (*n* = 53) were excluded from the study. The hazard ratios (HR) were standardized. *p* < 3.8 x 10^−3^ (0.05/13) was considered statistically significant (bold). *p* < 0.05 is considered as nominally significant (underlined). P unadjusted. P* adjusted for age. P** Adjusted for age and BMI. P*** Adjusted for age, BMI, smoking status, and reimbursement for hypertension. P**** Adjusted for age, BMI, smoking status, and reimbursement for hypertension and diabetes.

CI: confidence interval; CRP: C-reactive protein; HbA1c: hemoglobin A1c.

**Figure 2.**
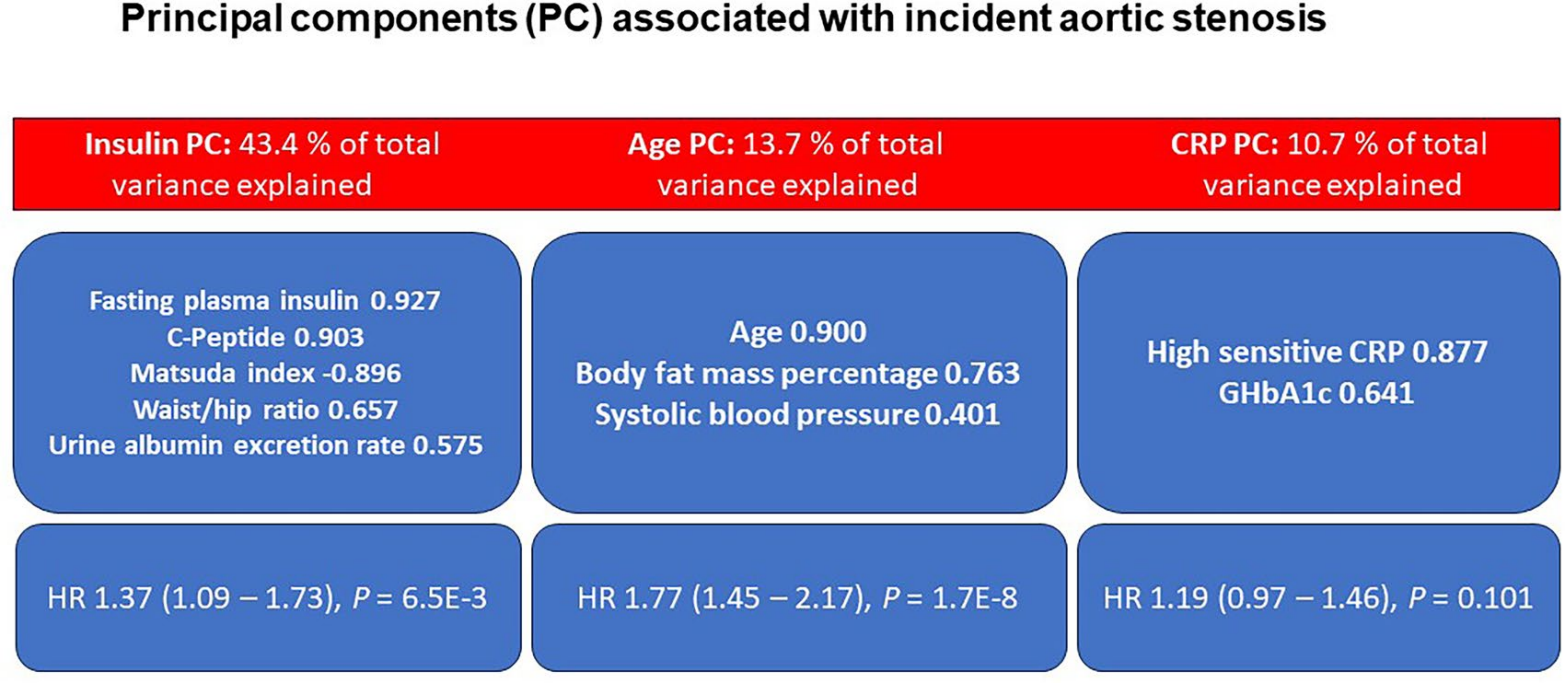
In a nonselected population of 10,144 Finnish men without aortic stenosis (as) at baseline, biomarkers associated with incident AS in unadjusted Cox regression analyses clustered into three principal components. Two of them were associated with incident AS with high or borderline statistical significance. HR: hazard ratio; PC: principal component.

Variables nominally or significantly associated with AS in unadjusted Cox regression analyses for all study subjects (see Tables 1 and 2) were entered into the principal component (PC) analysis. Of several measures of hyperinsulinemia, insulin resistance, glucose tolerance, body bioimpedance, waist circumference, and urine albumin, one parameter from each category was included in the PC analysis. Table 3 and Figure 2 show the Varimax rotated components, their loadings on the original biomarkers, and explained percentages of the total variance. Three components accounting for 67.8% of the total variance were identified. PC 1 consisted of fasting plasma insulin, serum C-peptide, Matsuda index, waist/hip ratio, and the urine albumin excretion rate. PC 2 consisted of age, body fat mass percentage, and systolic blood pressure, and PC 3 consisted of hs-CRP and GHbA1c. In Cox regression analysis, PC 1 and PC 2, but not PC3, were associated with incident AS with HRs of 1.37 and 1.77, respectively.

**Table 3.** Principal component analysis of baseline variables, which were associated with incident as in unadjusted Cox regression analyses, and unadjusted Cox regression analysis of the principal components associated with incident AS in the METSIM cohort.

Component		Factor loading	Percent variance explained	HR (95 % Cl)	*P*
PC 1	Fasting plasma insulin	**0.927**	43.4 %	1.37 (1.09-1.73)	6.5E-3
	C-Peptide	**0.903**			
	Matsuda index	**−0.896**			
	Waist/hip ratio	**0.657**			
	Urine albumin excretion rate	**0.575**			
PC 2	Age	**0.900**	13.7 %	1.77 (1.45-2.17)	**1.7E-8**
	Body fat mass percentage	**0.763**			
	Systolic blood pressure	**0.401**			
PC 3	High sensitive CRP GHbA1c	**0.877**	10.7 %	1.19 (0.97-1.46)	0.101
		**0.641**			

The analysis included 10,144 participants without AS at baseline, of whom 116 developed incident AS during the 10.8 yeas follow-up period. All variables except age were log-transformed for statistical analysis. The bold indicates variables with significant loadings (>0.400). P-value < 1.7 x 10^−3^ (0.05/3) was considered statistically significant (bold) and *p* < 0.05 is considered nominally significant (underlined).

CI: confidence interval; CRP: C-reactive protein; HbA1c: hemoglobin A1c.

Table S6 shows the univariate Cox regression analyses of the associations of baseline anthropometric factors and biomarkers with incident AS after excluding subjects with diabetes at baseline. Age, systolic blood pressure, body fat mass and fat-free mass percentages, fasting plasma insulin, OGTT 30 min plasma insulin and proinsulin, OGTT 120 min plasma proinsulin, serum C-peptide, and Matsuda index were significantly associated with incident AS in unadjusted Cox regression analyses. In addition, BMI, waist circumference, waist/hip ratio, height, body muscle mass percentage, urine albumin excretion rate, hs-CRP, OGTT 120 min plasma insulin, and fasting plasma proinsulin were nominally associated with incident AS. When adjusted for age, BMI, smoking status, and hypertension reimbursement, only systolic blood pressure remained nominally significant.

Table S**7** presents univariate Cox regression analyses of the associations of baseline anthropometric factors and biomarkers with incident AS in patients with a tricuspid aortic valve. Patients with AS and bicuspid aortic valve or an undefined number of cusps in the aortic valve were excluded from the analyses. In unadjusted Cox regression analyses, biomarkers significantly associated with AS were age, systolic blood pressure, height, body fat mass and fat-free mass percentages, hs-CRP, OGTT 30 min plasma insulin and proinsulin, OGTT 120 min plasma insulin and proinsulin, and Matsuda index. When adjusted for age, BMI, smoking status, reimbursement for hypertension and diabetes, systolic blood pressure, height, body muscle mass percentage, hs-CRP, 30 min plasma insulin and proinsulin, OGTT 120 min plasma proinsulin, and Matsuda index had nominal associations with incident AS.

After excluding subjects with diabetes at baseline examination, three components were identified in the PC analysis (Table S8), accounting for 66.4% of the total variance. The results were very similar to those of the PC analysis including all subjects with AS. Three PCs were found: PC 1, reflecting insulin resistance; PC 2, reflecting aging and hypertension; and PC 3, reflecting inflammation. In Cox regression analysis, PC 1 and PC 2 were significantly associated with AS with HRs 1.44 of 1.77.

Table S9 shows the PC analysis in subjects with incident AS, excluding those with bicuspid aortic valve and undefined number of cusps in the aortic valve. Three PCs, accounting for 69.2% of the total variance, were identified: PC 1, reflecting insulin resistance; PC 2, aging, obesity, and hypertension; PC 3, reflecting inflammation and declining kidney function. In Cox regression analyses, PC 1 and PC 2 were significantly associated with incident AS, with HRs 1.57 and 2.04, respectively.

For PC analyses in nondiabetic subjects, or when subjects with nontricuspid valves were excluded see supplementary data and Tables S8 and S9. Insulin resistance loaded on PC 1, which had a statistically significant association with incident AS.

## Discussion

### Principal findings

In the present large-scale population-based follow-up study, several biomarkers reflecting hyperinsulinemia and/or insulin resistance, including fasting, 30 min, and 120 min plasma insulin and proinsulin, Matsuda index, and serum C-peptide, were associated with incident AS, which is to the best of our knowledge a novel finding. Biomarkers of insulin resistance associated with incident AS, even after adjustments for BMI and hypertension, or exclusion of diabetic subjects or those with a nontricuspid aortic valve. In PC analyses, parameters related to hyperinsulinemia and insulin resistance were loaded on component 1, and age, fat mass, and systolic blood pressure were loaded on component 2. Both components predicted AS in the Cox regression analyses, suggesting that insulin resistance is an important risk factor for AS, independent of age, systolic blood pressure, diabetes, and obesity.

### In the context of current literature

#### Incidence of AS

In the present study, the incidence of AS was 1.1% during the follow-up of 10.8 years. Other large-scale studies report 0.1-1.6% incidence of AS for the respective follow-up time [8–12].

#### Previously identified risk factors of AS

Only a few longitudinal studies have investigated risk factors for AS. Recently, both systolic and diastolic blood pressures were reported to increase risk of AS in a cohort analysis of 5.4 million UK adults, confirming results of three previous population-based cohort studies [9]. Other previously identified risk factors of AS in prospective studies are age, waist, BMI, CRP, smoking, diabetes, dyslipidemia, LDL cholesterol and Lp(a) levels [8–13]. In the present study, of the aforementioned parameters, age, systolic blood pressure, waist, BMI, CRP, and diabetes but not diastolic blood pressure or smoking were associated with incident AS. Lp(a) was not measured in this study. LDL cholesterol levels were lower in subjects with incident AS than in those without AS. However, participants with incident AS used statins significantly more often than those who did not develop AS, probably biasing the interpretation of the analysis. Overall, most previously identified risk factors for AS were confirmed in the present study, validating the new finding that insulin resistance is a risk factor for AS. The PC analyses in our study showed that many of the risk factors for AS are interrelated and form three distinct clusters, compatible with multiple pathogenic processes in the development of AS.

#### NMR derived metabolites

To our knowledge, no previous study has investigated NMR-based metabolic measures as risk factors for AS. In the present study, only a few NMR-based metabolic measures were associated with incident AS, and the associations were nominally significant.

#### Diabetes and impaired fasting glucose and glucose tolerance

In the present study, diabetes increased the risk of AS, which is in accordance with previous studies [10, 11] Impaired fasting glucose and impaired glucose tolerance did not increase the risk of AS. To the best of our knowledge, there are no previous studies on the risk of AS in patients with impaired fasting glucose or glucose tolerance.

#### Hyperinsulinemia and insulin resistance

In the present study, several parameters measuring hyperinsulinemia and insulin resistance were found to be associated with incident AS. To the best of our knowledge, no previous prospective studies have investigated the association between hyperinsulinemia or insulin resistance and incident AS. In a Finnish cross-sectional study of an elderly population, fasting insulin was not frelated to aortic valve calcification or AS [21]. In a recent cross-sectional study, adipose tissue insulin resistance was associated with aortic valve calcification detected using computed tomography [22].

Metabolic syndrome, consisting of abdominal obesity, high blood pressure, low high-density lipoprotein (HDL) cholesterol, high triglycerides, and abnormal glucose tolerance, is characterized by insulin resistance. In two recent studies, metabolic syndrome and its components increased the risk of aortic valve calcium assessed by CT [23,24] However, direct measures of insulin resistance were not available, and high blood pressure, obesity, and diabetes, which are included in the definition of metabolic syndrome, further complicated the interpretation. Recently, a genetically increased body mass index was shown to be causally related to AS; however, even if insulin resistance is a common feature of obesity, no conclusions regarding the role of insulin resistance in AS can be deduced from the results of the study.

In the present study, several measures of insulin resistance and hyperinsulinemia associated with incident AS if diabetic subjects were excluded, suggesting that insulin is an important risk factor for AS in all subjects independent of diabetes. When subjects with AS in the nontricuspid valve were excluded, measures of insulin resistance associated with AS were even more significantly, suggesting that insulin resistance may be a particularly important risk factor for AS in subjects with a normal tricuspid aortic valve compared to those with congenitally malformed valves.

### Possible mechanisms

#### Hyperinsulinemia and insulin resistance

Insulin resistance increases the risk of several atherosclerotic vascular diseases [25–28]. Insulin resistance is already present within the normal range of fasting and 2 h plasma glucose levels [25], and insulin resistance has been shown to be a risk factor for atherosclerotic cardiovascular disease in both nondiabetic and diabetic subjects [25–29].

Insulin resistance induces atherogenic lipid metabolism, releases inflammatory proteins from the liver, increases blood pressure, decreases endothelial nitric oxide synthesis, promotes adhesion molecules in endothelial cells, and promotes apoptosis in macrophages [25]. Insulin resistance has been found to induce atherosclerotic calcification in a mouse model of atherosclerosis and the characteristics of type 2 diabetes [29]. In a recent study, hyperinsulinemia, in addition to hyperglycemia, led to the activation and differentiation of valvular interstitial cells of the aortic valve, matrix remodeling, and upregulation of smooth muscle actin expression and alkaline phosphatase activity, thereby altering the valve at the cellular level and showing amechanism by which insulin resistance and related hyperinsulinemia may contribute to the process of CAVD [30]. Thus, there are plausible mechanisms by which hyperinsulinemia induces CAVD, further confirming that AS is a form of atherosclerotic vascular disease for which hyperinsulinemia and insulin resistance have been documented as risk factors in several studies [25–29].

Age is an important risk factor for AS. In our study, at the baseline, the subjects with AS were older than those without AS. However, after adjusting for age (see Tables 1 and 2), the biomarkers reflecting insulin resistance remained highly statistically significant, showing that insulin resistance is related to AS independently of age. Moreover, in the PC analysis (Table 3, Figure 2), age and insulin resistance loaded on different components, indicating that they represent separate biomarkers for AS.

### Strengths and limitations

The strengths of this study include its large population-based cohort with a long follow-up period. The characteristics of the study participants were exceptionally comprehensive and detailed. At baseline, each study subject was phenotyped carefully, comprehensive data on the patient history of cardiovascular diseases and medications were collected, and a large panel of biomarkers was examined, including systemic metabolic measures determined by NMR. Insulin and glucose metabolism were examined comprehensively using several different parameters measuring insulin sensitivity, insulin secretion, and glucose tolerance, including the Matsuda index and insulin and proinsulin measurements in the oral glucose tolerance test. The main limitations of this study were the inclusion of only male subjects and a relatively small number of incident AS cases.

### Clinical implications

Insulin resistance is extremely common in Western populations [25]. As insulin resistance and hyperinsulinemia appear to be important risk factors for AS, measures improving insulin sensitivity, such as weight control and exercise, might be effective in the prevention of aortic stenosis.

## Conclusions

Biomarkers reflecting insulin resistance are risk factors for AS independent of other cardiovascular risk factors, such as age, diabetes, obesity, and systolic blood pressure, a novel finding indicating that insulin resistance is important in the pathogenesis of aortic stenosis. If measures increasing insulin sensitivity decrease the risk of aortic stenosis warrants further studies.

## Supplementary Material

Supplemental Material

## Data Availability

The data that support the findings of this study are available from the corresponding author JK, upon reasonable request.
