# Supplementary material for: Biomarkers reflecting insulin resistance increase the risk of aortic stenosis in a population-based study of 10,144 Finnish men
Source: Ann Med. 2024 Nov 26;56(1):2419996. doi: 10.1080/07853890.2024.2419996 (PMC11610258; doi:10.1080/07853890.2024.2419996)
Supplement: Supplemental Material [file IANN_A_2419996_SM3931.zip › Suppl_Data/Supplementary Information Biomarkers of Aortic stenosis final.docx]

**SUPPLEMENTARY INFORMATION**

**METHODS**

**Anthropometric measurements** **at baseline**

Height and weight were measured to the nearest 0.5 cm and 0.1 kg, respectively. Body mass index (BMI) was calculated as weight (kg) divided by height (m) squared. Waist circumference was measured at the midpoint between the lateral iliac crest and lowest rib. Blood pressure (BP) was measured in the sitting position after a 5-min rest with a mercury sphygmomanometer. The average of three measurements was used to calculate systolic and diastolic BP. Smoking status was defined as current smoking (yes vs no). Diagnosis of hypertension was based on the use of antihypertensive medication. Information of medications was obtained from the Finnish National Drug Reimbursement registry.

**Oral glucose tolerance test at METSIM baseline**

A 2 h oral glucose tolerance test (OGTT) (75 g of glucose) was performed, and samples for plasma glucose insulin, and proinsulin were drawn at 0, 30, and 120 min. Glucose tolerance based on an OGTT was classified according to ADA 2003 criteria, including type 2 diabetes (fasting glucose tolerance test ≥7.0 mmol/l and/or 2 h plasma glucose ≥11.1 mmol/l), impaired fasting glucose (IFG) (fasting plasma glucose levels 5.6 mmol/l to 6.9 mmol/l), and impaired glucose tolerance (IGT) (2-h values in OGTT of 7.8 mmol/l to 11.0 mmol/l).^1^

**C-peptide and Matsuda index**

Matsuda index of insulin sensitivity (ISI) was calculated to estimate insulin sensitivity.^2^ Plasma C-peptide concentrations were measured by commercial chemiluminescence assays (DiaSorin Liaison®Analyzer, DiaSorin Deutchland GmbH, Dietzenbach, Germany).

**Diagnosis of diabetes**

The diagnosis of type 2 diabetes was based on the following: fasting plasma glucose (FPG) ≥7.0 mmol/L, 2-hour plasma glucose (2hPG) level in an OGTT of ≥11.1 mmol/L or glycated hemoglobin (HbA1c) of ≥6.5%, or type 2 diabetes diagnosed by a physician according to the medical records obtained from the National Drug Reimbursement registry.^3^ The diagnosis of type 1 diabetes was based on the diagnosis by a physician according to the medical records.

**Diagnosis of previous cardiovascular disease (CVD) events**

CVD events and procedures were defined according to the internationally accepted ICD -10 criteria and identified from the Kuopio University Hospital medical records**.**

**Laboratory measurements**

Levels of plasma glucose, insulin, proinsulin, hemoglobin A1c (HbA1c), total triglycerides, plasma free fatty acids, high-density lipoprotein (HDL) cholesterol, and low-density lipoprotein cholesterol, alanine aminotransferase, apolipoproteins A1 and B (ApoA1 and ApoB), glycoprotein acetyls, glomerular filtration rate, and urinary albumin were measured as previously described.^3,4^

**Inflammatory biomarkers**

Serum concentrations of high sensitive CRP (hs-CRP) were assayed using kinetic immunoturbidimetry (near-infrared particle immunoassay; IMMAGE Immunochemistry System; Beckman Coulter, Fullerton, CA) and plasma interleukin 1 receptor antagonist (IL-1RA) using a photometric immunoassay [enzyme-linked immunosorbent assay (ELISA)] method (Quantikine DRA00 Human IL-1RA; R&D Systems, Inc., Minneapolis, MN). Plasma adiponectin was measured using ELISA (human adiponectin ELISA kit; Linco Research, St. Charles, MI). Concentrations of glycoprotein acetyls (including mainly alpha-1-acid glycoprotein) were quantified from serum samples using a high-throughput proton nuclear magnetic resonance metabolomics platform, as previously described.^5^

**Proton nuclear magnetic resonance analysis**

For all METSIM study participants, proton NMR analysis was performed at the same time from the baseline serum samples. Altogether, 27 serum metabolic measures, including amino acids, fatty acids, ketone bodies, and lipoprotein lipids (Supporting Information, Table S5), were determined using a high-throughput serum NMR platform operating at 500 MHz. Fasting serum samples collected at baseline were stored at 80°C and thawed overnight in a refrigerator before sample preparation. Aliquots of each sample (300 μL) were mixed with sodium phosphate buffer (300 μL). The NMR data were measured using a Bruker AVANCE III spectrometer operating at 500.36 MHz (1 H observation frequency; 11.74 T) and equipped with an inverse selective SEI probehead, including an automatic tuning and matching unit and a z-axis gradient coil for automated shimming. A BTO-2000 thermocouple serves to temperature stabilization, the sample at the level of approximately 0.01°C. The NMR technology is widely used, standardized, and accredited (https://nightingalehealth.com/ about/technology). Details of the NMR experimentation have been described previously.^6,7^

**REFERENCES OF METHODS**

1. American Diabetes Association. Diagnosis and classification of diabetes mellitus. *Diabetes Care* 2013;36(Suppl 1):67.

2. M Matsuda, RA DeFronzo. Insulin sensitivity indices obtained from oral glucose tolerance testing: Comparison with the euglycemic insulin clamp. *Diabetes Care* 1999;9:1462-1470.

3. Stancakova A, Javorsky M, Kuulasmaa T, Haffner SM, Kuusisto J, Laakso M. Changes in insulin sensitivity and insulin release in relation to glycemia and glucose tolerance in 6,414 Finnish men. *Diabetes* 2009;58:1212-1221.

4. Laakso M, Kuusisto J, Stancakova A, *et al*. The metabolic syndrome in men study: A resource for studies of metabolic and cardiovascular diseases. *J Lipid Res* 2017;58:481-493.

5. Fizelova M, Jauhiainen R, Kangas AJ*, et al*. Differential associations of inflammatory markers with insulin sensitivity and secretion: The prospective METSIM study. *J Clin Endocrinol Metab* 2017;102:3600-3609.

6. Soininen P, Kangas AJ, Würtz P, Suna T, Ala-Korpela M. Quantitative serum nuclear magnetic resonance metabolomics in cardiovascular epidemiology and genetics. *Circ Cardiovasc Genet* 2015;8:192-206.

7. Inouye M, Kettunen J, Soininen P, *et al*. Metabonomic, transcriptomic, and genomic variation of a population cohort. *Mol Syst Biol* 2010;6:441.
