# Supplementary material for: Biomarkers reflecting insulin resistance increase the risk of aortic stenosis in a population-based study of 10,144 Finnish men
Source: Ann Med. 2024 Nov 26;56(1):2419996. doi: 10.1080/07853890.2024.2419996 (PMC11610258; doi:10.1080/07853890.2024.2419996)
Supplement: Supplemental Material [file IANN_A_2419996_SM3931.zip › Suppl_Data/Supplementary Tables Ann_Med.docx]

|  |  | N  Case | | **Incident AS**  (N=116)  **Mean ± SD or**  **n (%)** |  |  |
| --- | --- | --- | --- | --- | --- | --- |
| **Symptoms related to AS** |  |  | | |  |  |
| NYHA classification |  |  | | |  |  |
| I | | | |  | 77 (66.4) |  |
| II | | | |  | 28 (24.1) |  |
| III | | | |  | 11 (9.5) |  |
| IV | | | |  | 0 (0.0) |  |
| Syncope | | |  | 3 (2.6) |  |  |
| Angina pectoris | | |  | 32 (27.6) |  |  |
| Clinical heart failure | | |  | 12 (10.3) |  |  |
| **Heart rhythm** | | | 96 |  |  |  |
| Sinus rhythm | | |  | 86 (89.6) |  |  |
| Atrial fibrillation | | |  | 9 (9.4) |  |  |
| Pacemaker rhythm | | |  | 1 (1.0) |  |  |
| **QRS-complex morphology in ECG** | | | 97 |  |  |  |
| Normal | | |  | 35 (36.1) |  |  |
| Left ventricular hypertrophy | | |  | 36 (37.1) |  |  |
| Left bundle branch block | | |  | 4 (4.1) |  |  |
| Right bundle branch block | | |  | 6 (6.2) |  |  |
| Intraventricular conduction delay | | |  | 15 (15.5) |  |  |
| Pacemaker | | |  | 1 (0.9) |  |  |
| QRS-complex duration [ms] | | | 94 | 106.5 ± 25.0 |  |  |
| **Echocardiographic** **parameter** | | |  |  |  |  |
| Left ventricular end-diastolic diameter [mm] | | | 109 | 54.0 ± 6.5 |  |  |
| Left ventricular end-systolic diameter [mm] | | | 91 | 35.5 ± 8.5 |  |  |
| End-diastolic interventricular septum thickness [mm] | | | 106 | 12.9 ± 2.6 |  |  |
| End-diastolic posterior wall thickness [mm] | | | 99 | 11.6 ± 1.9 |  |  |
| Ejection fraction [%] | | | 108 | 60.6 ± 12.2 |  |  |
| Aortic root diameter [mm] | | | 102 | 35.9 ± 4.6 |  |  |
| Left atrial dimension [mm] | | | 100 | 41.8 ± 7.0 |  |  |
| Aortic valve maximal gradient [mmHg] | | | 116 | 35.5 ± 18.4 |  |  |
| Number of cusps in aortic valve | | |  |  |  |  |
| Tricuspid | | |  | 73 (62.9) |  |  |
| Bicuspid | | |  | 22 (19.0) |  |  |
| Impossible to define or not mentioned | | |  | 21 (18.1) |  |  |
| **Aortic regurgitation** | | |  | 59 (50.1) |  |  |
| Gradus 1 | | |  | 34 (57.6) |  |  |
| Gradus 2 | | |  | 16 (27.1) |  |  |
| Gradus 3 | | |  | 8 (13.6) |  |  |
| Gradus 4 | | |  | 1 (1.7) |  |  |

**Table S1** Clinical, ECG and echocardiographic characteristics of the METSIM study participants with incident aortic stenosis (AS) at the time of the diagnosis of AS.

NYHA classification = New York Heart Association Classification

**Table S2** METSIM Study baseline clinical characteristics of the study participants without and with incident aortic stenosis (AS). Individuals with AS at baseline (n=53) are excluded.

|  | All participants | |  |
| --- | --- | --- | --- |
|  | (N=10,144) | |  |
|  | No AS  (N=10,028)  Mean ± SD or  n (%) | Incident AS  (N=116)  Mean ± SD or  n (%) | *P* |
| **Conventional risk factors** |  |  |  |
| Age [y] | 57.6 ± 7.1 | 61.9 ± 6.5 | **5.8E-11** |
| Current smokers | 1812 (18.1) | 21 (18.1) | 0.992 |
| Systolic blood pressure [mmHg] | 138.2 ± 16.7 | 146.3 ± 19.4 | **3.8E-7** |
| Diastolic blood pressure [mmHg] | 87.3 ± 9.4 | 87.2 ± 10.5 | 0.863 |
| Height [cm] | 175.9 ± 6.3 | 174.2 ± 6.9 | 2.9E-3 |
| Weight [kg] | 84.5 ± 14.1 | 86.5 ± 14.4 | 0.117 |
| Body mass index [kg/m^2^] | 27.3 ± 4.2 | 28.5 ± 4.4 | **2.0E-3** |
| Waist [cm] | 98.7 ± 11.5 | 102.2 ± 11.8 | **1.1E-3** |
| Waist/hip ratio | 0.97 ± 0.1 | 1.00 ± 0.1 | **5.9E-4** |
| Diabetes* | 1,393 (13.9) | 30 (25.9) | **2.3E-4** |
| History of myocardial infarction | 395 (3.9) | 5 (4.3) | 0.826 |
| History of stroke | 191 (1.9) | 5 (4.3) | 0.834 |
| History of coronary bypass operation | 341 (3.4) | 7 (6.0) | 0.121 |
| History of coronary artery angioplasty | 297 (3.0) | 3 (2.6) | 0.812 |
| History of heart failure | 66 (0.7) | 4 (3.4) | **3.1E-5** |
| History of leg artery revascularisation | 53 (0.5) | 3 (2.6) | 2.9E-3 |
| History of carotid artery operation | 28 (0.3) | 2 (1.7) | 4.4E-3 |
|  |  |  |  |
| **Bioimpedance** |  |  |  |
| Fat mass (%) | 24.0 ± 6.5 | 27.7 ± 7.3 | **3.5E-8** |
| Muscle mass (%) | 43.6 ± 5.6 | 42.1 ± 6.4 | 3.2E-3 |
| Fat free mass (%) | 76.0 ± 6.5 | 72.3 ± 7.3 | **9.6E-10** |

P < 2.5 x 10^-3^ (0.05/20) is considered statistically significant (bold). P<0.05 is considered nominally significant (underlined). All variables except for age were log-transformed to correct for skewed distribution. P Independent Samples T-test or Pearson Chi-Square Test.

*Diabetes in the baseline measurements (includes newly and previously diagnosed subjects).

**Table S3** METSIM Study baseline metabolic and inflammatory biomarkers of the study participants without and with incident aortic stenosis (AS). Individuals with AS at baseline (n=53) are excluded.

|  | All participants | |  |
| --- | --- | --- | --- |
|  | (N=10,144) | |  |
|  | No AS  (N = 10,028)  Mean ± SD | IncidentAS  (N=116)  Mean ± SD | *P* |
| Total cholesterol [mmol/l | 5.31 ± 1.03 | 5.05 ± 0.98 | 7.9E-3 |
| LDL cholesterol [mmol/l] | 3.31 ± 0.90 | 3.12 ± 0.89 | 0.018 |
| HDL cholesterol [mmol/l] | 1.44 ± 0.40 | 1.41 ± 0.40 | 0.337 |
| Total triglycerides [mmol/l] | 1.47 ± 1.02 | 1.47 ± 0.79 | 0.503 |
| ApoA1 [g/l] | 1.41 ± 0.24 | 1.39 ± 0.24 | 0.403 |
| ApoB [g/l] | 1.04 ± 0.28 | 1.02 ± 0.29 | 0.371 |
| ALT [U/l] | 32.5 ± 21.3 | 33.3 ± 23.8 | 0.956 |
| Creatinine [mmol/l] | 83.9 ± 15.4 | 84.0 ± 11.6 | 0.816 |
| eGFR MDRD | 89.5 ± 15.0 | 87.7 ± 14.6 | 0.255 |
| eGFR Cockroft-Gault | 104.6 ± 26.6 | 101.1 ± 25.7 | 0.150 |
| Urine albumin [mg/l] | 29.1 ± 164.3 | 42.7 ± 125.5 | **1.9E-3** |
| Urine albumin excretion rate [ug/min] | 23.5 ± 139.3 | 51.1 ± 285.7 | **1.3E-3** |
| Plasma adiponectin [ug/ml] | 7.8 ± 4.4 | 7.9 ± 4.2 | 0.897 |
| High sensitivity CRP [mg/l] | 2.2 ± 4.5 | 2.8 ± 3.8 | **1.1E-3** |
| IL1 receptor antagonist [pg/ml] | 228.3 ± 194.1 | 240.2 ± 187.4 | 0.244 |
| IL1 beta [pg/ml] | 0.33 ± 0.69 | 0.34 ± 0.54 | 0.478 |
| Glycoprotein acetyls [mmol/l] | 1.38 ± 0.24 | 1.40 ± 0.21 | 0.230 |

P < 2.9 x 10^-3^ (0.05/17) is considered statistically significant (bold). P<0.05 is considered nominally significant (underlined). All variables were log-transformed to correct for skewed distribution. P Independent Samples T-test.

IL1, interleukin-1

|  | All participants  (N=10,144) | |  |
| --- | --- | --- | --- |
|  |  |  |  |
|  | No AS  (N=10,028)  Mean ± SD or  n (%) | Incident AS  (N=116)  Mean ± SD or  n (%) | ***P*** |
| Fasting plasma glucose [mmol/l] | 5.97 ± 1.11 | 6.18 ± 1.23 | 0.028 |
| OGTT 30 min plasma glucose [mmol/l] | 9.00 ± 1.69 | 9.33 ± 1.75 | 0.066 |
| OGTT 120 min plasma glucose [mmol/l] | 6.45 ± 2.40 | 6.85 ± 2.71 | 0.174 |
| Blood HbA1c [%] | 5.80 ± 0.62 | 5.96 ± 0.76 | 4.5E-3 |
| Fasting plasma insulin [mU/l] | 9.85 ± 12.84 | 14.48 ± 18.15 | **6.1E-6** |
| OGTT 30 min plasma insulin [mU/L] | 66.55 ± 48.78 | 82.53 ± 56.96 | **7.1E-4** |
| OGTT 120 min plasma insulin [mU/L] | 55.62 ± 57.57 | 72.71 ± 65.74 | **2.3E-3** |
| Fasting plasma proinsulin [mU/L] | 15.33 ± 9.99 | 19.64 ± 14.94 | **5.6E-5** |
| OGTT 30 min plasma proinsulin [mU/L] | 31.38 ± 15.71 | 37.03 ± 20.27 | **9.1E-4** |
| OGTT 120 min plasma proinsulin [mU/L] | 52.68 ± 28.13 | 61.43 ± 35.84 | **3.5E-3** |
| Serum C-peptide [nmol/l] | 0.51 ± 0.24 | 0.61 ± 0.31 | **8.9E-5** |
| Matsuda index [mg/dl, mU/l] | 6.68 ± 4.16 | 5.29 ± 3.57 | **8.7E-5** |

**Table S4** METSIM Study baseline glucose and insulin metabolism biomarkers of the study participants without and with incident aortic stenosis (AS). Individuals with AS at baseline (n=53) are excluded.

P < 4.2 x 10^-3^ (0.05/12) is considered statistically significant (bold). P <0.05 is considered nominally significant (underlined). P, Independent Samples T-test. All variables were log-transformed to correct for skewed distribution.

OGTT, oral glucose tolerance test.

|  | No AS  (N=10,028)  Mean ± SD | Incident AS  (N=116)  Mean ± SD | ***P*** |
| --- | --- | --- | --- |
|  |  |  |  |
| **Amino acids** |  |  |  |
| Alanine [mmol/l] | 0.43 ± 0.07 | 0.43 ± 0.06 | 0.339 |
| Glutamine [mmol/l] | 0.52 ± 0.08 | 0.51 ± 0.08 | 0.095 |
| Glycine [mmol/l] | 0.27 ± 0.04 | 0.27 ± 0.05 | 0.693 |
| Histidine [g/l] | 0.07 ± 0.01 | 0.07 ± 0.01 | 0.050 |
| Isoleucine [mmol/l] | 0.06 ± 0.02 | 0.06 ± 0.02 | 0.695 |
| Leucine [mmol/l] | 0.09 ± 0.02 | 0.09 ± 0.02 | 0.878 |
| Phenylalanine [mmol/l] | 0.08 ± 0.01 | 0.08 ± 0.01 | 0.028 |
| Tyrosine [mmol/l] | 0.06 ± 0.01 | 0.06 ± 0.01 | 0.106 |
| Valine [mmol/l] | 0.22 ± 0.04 | 0.22 ± 0.04 | 0.336 |
|  |  |  |  |
| **Fatty acids** |  |  |  |
| Fasting plasma FFA [mmol/l] | 0.38 ± 0.16 | 0.41 ± 0.18 | 0.039 |
| OGTT 30 min plasma FFA [mmol/l] | 0.25 ± 0.12 | 0.26 ± 0.13 | 0.273 |
| OGTT 120 min plasma FFA [mmol/l] | 0.05 ± 0.04 | 0.05 ± 0.03 | 0.704 |
| Plasma FFA under the curve (OGTT) [mmol/l*min) | 22.9 ± 10.1 | 24.3 ± 10.6 | 0.180 |
| Omega-3 fatty acids [mmol/l] | 0.70 ± 0.22 | 0.72 ± 0.25 | 0.531 |
| Ratio of omega-3 fatty acids to total fatty acids [%] | 5.15 ± 1.30 | 5.45 ± 1.68 | 0.155 |
| Omega-6 fatty acids [mmol/l] | 4.19 ± 0.89 | 4.03 ± 0.90 | 0.033 |
| Ratio of omega-6 fatty acids to total fatty acids [%] | 30.7 ± 3.4 | 30.3 ± 3.4 | 0.225 |
| Total fatty acids [mmol/l] | 13.8 ± 3.4 | 13.4 ± 3.2 | 0.174 |
| Saturated fatty acids [mmol/l] | 5.23 ± 1.47 | 5.06 ± 1.31 | 0.135 |
| Ratio of saturated fatty acids to total fatty acids [%] | 37.7 ± 2.3 | 37.5 ± 2.3 | 0.288 |
| Estimated degree of unsaturation | 1.19 ± 0.08 | 1.19 ± 0.09 | 0.227 |
| 18:2, linoleic acid [mmol/l] | 3.29 ± 0.71 | 3.15 ± 0.74 | 0.549 |
| Ratio of 18:2, linoleic acid to total fatty acids [%] | 24.1 ± 3.3 | 23.6 ± 3.4 | 0.838 |
| Other PUFAs [mmol/l] | 4.89 ± 1.03 | 4.76 ± 1.02 | 0.112 |
| Ratio of PUFAs to total fatty acids [%] | 35.8 ± 3.8 | 35.7 ± 3.7 | 0.817 |
| 22:6, docosahexaenoic acid [mmol/l] | 0.22 ± 0.08 | 0.23 ± 0.09 | 0.657 |
| Ratio of 22:6, docosahexaenoic acid to total fatty acids [%] | 1.64 ± 0.52 | 1.74 ± 0.64 | 0.141 |
| MUFAs; 16:1, 18:1 [mmol/l] | 3.68 ± 1.24 | 3.62 ± 1.15 | 0.529 |
| Ratio of MUFAs to total fatty acids [%] | 26.5 ± 3.5 | 26.8 ± 3.5 | 0.336 |
| **Lipids** |  |  |  |
| Total phosphoglyserides [mmol/l] | 2.26 ± 0.47 | 2.17 ± 0.47 | 0.020 |
| Ratio of triglyserides to phosphoglyserides [mmol/l] | 0.67 ± 0.26 | 0.71 ± 0.23 | 0.057 |
| Sphingomyelins [mmol/l] | 0.57 ± 0.14 | 0.55 ± 0.15 | 0.257 |
| Total cholines [mmol/l] | 2.70 ± 0.50 | 2.62 ± 0.50 | 0.063 |
| PC and other cholines [mmol/l] | 2.19 ± 0.41 | 2.13 ± 0.412 | 0.075 |
| ApoA1 [g/l] | 1.56 ± 0.24 | 1.51 ± 0.22 | 0.038 |
| ApoB [g/l] | 1.04 ± 0.25 | 1.00 ± 0.25 | 0.056 |
| Ratio of ApoB to ApoA1 | 0.68 ± 0.17 | 0.67 ± 0.17 | 0.570 |
| **Ketone bodies** |  |  |  |
| Acetate [mmol/l] | 0.04 ± 0.04 | 0.04 ± 0.01 | 0.794 |
| Acetoacetate [mmol/l] | 0.06 ± 0.05 | 0.06 ± 0.05 | 0.340 |
| 3-hydroxybutyrate [mmol/l] | 0.16 ± 0.10 | 0.17 ± 0.08 | 0.041 |
| **Energy metabolites** |  |  |  |
| Lactate [mmol/l] | 1.86 ± 0.49 | 1.89 ± 0.56 | 0.621 |
| Citrate [mmol/l] | 0.11 ± 0.02 | 0.11 ± 0.02 | 0.115 |
| Glycerol [mmol/l] | 0.07 ± 0.02 | 0.08 ± 0.03 | 0.274 |
| Pyruvate [mmol/l] | 0.07 ± 0.02 | 0.07 ± 0.03 | 0.222 |

**Table S5** METSIM Study baseline serum metabolic measures in proton NMR analysis a in the participants with and without incident aortic stenosis (AS) during the METSIM study follow-up. Individuals with AS at the baseline (n=53) are excluded.

P-value 1.1 x 10^-4^ (0.05/44) is considered statistically significant. P-value <0.05 is considered nominally significant (underlined). OGTT, oral glucose tolerance test; PC, phosphatidylcholine; PUFA, polyunsaturated fatty acid; MUFA, monounsaturated fatty acid.

P Independent Samples T-test.

**Supplementary table S6** Cox regression analysis of anthropometric and metabolic biomarkers as predictors for incident AS in 8710 participants, of whom 86 participants developed AS during the 10.8-year follow-up period. Participants with diabetes (n=1423) or AS (n=53) at baseline are excluded.

|  |  |  | HR |  | 95 %Cl | | | | *P* |  | *P** |  | *P*** |  | *P**** |  |
| --- | --- | --- | --- | --- | --- | --- | --- | --- | --- | --- | --- | --- | --- | --- | --- | --- |
|  | N  NON-CASE/CASE |  |  |  | Lower |  | Upper | |  |  |  |  |  |  |  |  |
| **Conventional risk factors** |  |  |  |  |  |  |  |  |  |  |  |  |  |  |  |  |
| Age | 8624/86 |  | 1.81 |  | 1.44 |  | 2.28 |  | **4.0E-7** |  |  |  |  |  |  |  |
| Systolic blood pressure | 8624/86 |  | 1.57 |  | 1.28 |  | 1.93 |  | **1.3E-5** |  | 1.6E-3 |  | 6.2E-3 |  | 8.3E-3 |  |
| Body mass index | 8621/86 |  | 1.37 |  | 1.11 |  | 1.69 |  | 3.2E-3 |  | 2.3E-3 |  |  |  |  |  |
| Waist | 8620/86 |  | 1.37 |  | 1.11 |  | 1.70 |  | 3.6E-3 |  | 5.8E-3 |  | 0.912 |  | 0.972 |  |
| Waist/hip ratio | 8619/86 |  | 1.30 |  | 1.05 |  | 1.62 |  | 0.019 |  | 0.053 |  | 0.950 |  | 0.779 |  |
| Height | 8621/86 |  | 0.79 |  | 0.64 |  | 0.97 |  | 0.023 |  | 0.345 |  | 0.390 |  | 0.438 |  |
|  |  |  |  |  |  |  |  |  |  |  |  |  |  |  |  |  |
| **Bioimpedance** |  |  |  |  |  |  |  |  |  |  |  |  |  |  |  |  |
| Body fat mass percentage | 8603/85 |  | 1.69 |  | 1.34 |  | 2.13 |  | **8.2E-6** |  | 0.051 |  | 0.987 |  | 0.955 |  |
| Body muscle mass percentage | 8603/85 |  | 0.80 |  | 0.66 |  | 0.98 |  | 0.030 |  | 0.948 |  | 0.230 |  | 0.200 |  |
| Body fat free mass percentage | 8603/85 |  | 0.62 |  | 0.51 |  | 0.75 |  | **5.2E-7** |  | 0.021 |  | 0.683 |  | 0.747 |  |
|  |  |  |  |  |  |  |  |  |  |  |  |  |  |  |  |  |
| **Blood biomarkers** |  |  |  |  |  |  |  |  |  |  |  |  |  |  |  |  |
| Urine albumin | 8541/86 |  | 1.20 |  | 0.98 |  | 1.47 |  | 0.077 |  | 0.310 |  | 0.552 |  | 0.706 |  |
| Urine albumin excretion rate | 8537/86 |  | 1.23 |  | 1.00 |  | 1.51 |  | 0.048 |  | 0.081 |  | 0.175 |  | 0.213 |  |
| High sensitive CRP | 8623/85 |  | 1.35 |  | 1.10 |  | 1.67 |  | 4.2E-3 |  | 8.1E-3 |  | 0.066 |  | 0.107 |  |
| Fasting plasma glucose | 8624/86 |  | 1.20 |  | 0.83 |  | 1.74 |  | 0.337 |  | 0.407 |  | 0.892 |  | 0.956 |  |
| Blood HbA1c | 8602/86 |  | 1.28 |  | 0.91 |  | 1.79 |  | 0.162 |  | 0.811 |  | 0.792 |  | 0.648 |  |
| Fasting plasma insulin | 8620/86 |  | 1.50 |  | 1.19 |  | 1.88 |  | **5.1E-4** |  | **5.0E-4** |  | 0.039 |  | 0.052 |  |
| OGTT 30 min plasma insulin | 8580/86 |  | 1.38 |  | 1.12 |  | 1.71 |  | **2.3E-3** |  | 3.0E-3 |  | 0.064 |  | 0.074 |  |
| OGTT 120 min plasma insulin | 8615/86 |  | 1.33 |  | 1.07 |  | 1.66 |  | 0.010 |  | 0.072 |  | 0.507 |  | 0.520 |  |
| Fasting plasma proinsulin | 8622/86 |  | 1.41 |  | 1.12 |  | 1.78 |  | 3.3E-3 |  | 4.0E-3 |  | 0.121 |  | 0.155 |  |
| OGTT 30 min plasma proinsulin | 8589/86 |  | 1.37 |  | 1.11 |  | 1.70 |  | **3.5E-4** |  | 3.0E-3 |  | 0.054 |  | 0.068 |  |
| OGTT 120 min plasma proinsulin | 8618/86 |  | 1.34 |  | 1.08 |  | 1.67 |  | **7.9E-4** |  | 0.038 |  | 0.284 |  | 0.314 |  |
| Serum C-peptide | 8584/85 |  | 1.56 |  | 1.23 |  | 1.97 |  | **2.6E-4** |  | **1.3E-3** |  | 0.039 |  | 0.067 |  |
| Matsuda index | 8572/86 |  | 0.67 |  | 0.56 |  | 0.85 |  | **5.1E-4** |  | **1.5E-3** |  | 0.081 |  | 0.102 |  |

CRP, C-reactive protein; HbA1c, haemoglobin A1c; OGTT, oral glucose tolerance test

Analysis includes 10,101 participants without incident AS at baseline, of whom 73 participants developed incident AS during the follow-up period. Hazard ratios (HR) are standardized. P < 2.3 x 10^-3^ (0.05/22) is considered statistically significant (bold). P<0.05 is considered as nominally significant (underlined).

P unadjusted. P* adjusted for age. P** adjusted for age and BMI. P*** Adjusted for age, BMI, smoking status and reimbursement for hypertension.

**Supplementary table S7** Cox regression analysis of anthropometric and metabolic biomarkers as predictors for incident AS in 10,101 participants of whom 73 participants developed AS during the 10.8-year follow-up period. Participants with AS at baseline (n=53) and AS patients with bicuspid aortic valve (n=22) or undefined number of cusps in aortic valve (n=21) are excluded.

|  |  |  | HR |  | 95 %Cl | | | | *P* |  | *P** |  | *P*** |  | *P**** |  |
| --- | --- | --- | --- | --- | --- | --- | --- | --- | --- | --- | --- | --- | --- | --- | --- | --- |
|  | N  NON-CASE/CASE |  |  |  | Lower |  | Upper | |  |  |  |  |  |  |  | *P***** |
| **Conventional risk factors** |  |  |  |  |  |  |  |  |  |  |  |  |  |  |  |  |
| Age | 10,028/73 |  | 2.24 |  | 1.74 |  | 2.89 |  | **5.3E-10** |  |  |  |  |  |  |  |
| Systolic blood pressure | 10,027/73 |  | 1.52 |  | 1.22 |  | 1.88 |  | **1.8E-4** |  | 0.022 |  | 0.054 |  | 0.051 | 0.047 |
| Body mass index | 10,025/73 |  | 1.30 |  | 1.05 |  | 1.60 |  | 0.016 |  | 0.014 |  |  |  |  |  |
| Waist | 10,023/73 |  | 1.29 |  | 1.04 |  | 1.60 |  | 0.021 |  | 0.044 |  | 0.649 |  | 0.522 | 0.546 |
| Waist/hip ratio | 10,022/73 |  | 1.41 |  | 1.13 |  | 1.79 |  | 2.5E-3 |  | 0.016 |  | 0.287 |  | 0.397 | 0.371 |
| Height | 10,025/73 |  | 0.64 |  | 0.51 |  | 0.80 |  | **9.6E-5** |  | 0.024 |  | 0.029 |  | 0.031 | 0.031 |
|  |  |  |  |  |  |  |  |  |  |  |  |  |  |  |  |  |
| **Bioimpedance** |  |  |  |  |  |  |  |  |  |  |  |  |  |  |  |  |
| Body fat mass percentage | 10,005/72 |  | 1.93 |  | 1.49 |  | 2.49 |  | **5.0E-7** |  | 0.069 |  | 0.765 |  | 0.836 | 0.841 |
| Body muscle mass percentage | 10,005/72 |  | 0.87 |  | 0.70 |  | 1.07 |  | 0.187 |  | 0.233 |  | 0.018 |  | 0.014 | 0.015 |
| Body fat free mass percentage | 10,005/72 |  | 0.59 |  | 0.49 |  | 0.72 |  | **9.8E-8** |  | 0.067 |  | 0.827 |  | 0.901 | 0.904 |
|  |  |  |  |  |  |  |  |  |  |  |  |  |  |  |  |  |
| **Blood biomarkers** |  |  |  |  |  |  |  |  |  |  |  |  |  |  |  |  |
| Urine albumin | 9888/73 |  | 1.18 |  | 0.98 |  | 1.42 |  | 0.077 |  | 0.521 |  | 0.910 |  | 0.951 | 0.990 |
| Urine albumin excretion rate | 9882/73 |  | 1.20 |  | 1.00 |  | 1.45 |  | 0.057 |  | 0.200 |  | 0.449 |  | 0.501 | 0.440 |
| High sensitive CRP | 10,026/73 |  | 1.43 |  | 1.15 |  | 1.78 |  | **1.4E-3** |  | 2.5E-3 |  | 0.017 |  | 0.033 | 0.031 |
| Fasting plasma glucose | 10,028/73 |  | 1.07 |  | 0.84 |  | 1.29 |  | 0.715 |  | 0.872 |  | 0.351 |  | 0.323 | 0.879 |
| Blood HbA1c | 10,005/73 |  | 1.06 |  | 0.85 |  | 1.31 |  | 0.607 |  | 0.441 |  | 0.149 |  | 0.810 | 0.103 |
| Fasting plasma insulin | 10,024/73 |  | 1.35 |  | 1.11 |  | 1.66 |  | 3.2E-3 |  | 8.4E-3 |  | 0.156 |  | 0.177 | 0.131 |
| OGTT 30 min plasma insulin | 9259/63 |  | 1.65 |  | 1.30 |  | 2.10 |  | **4.5E-5** |  | **6.0E-5** |  | 2.9E-3 |  | 3.4E-3 | 5.7E-3 |
| OGTT 120 min plasma insulin | 9295/63 |  | 1.52 |  | 1.18 |  | 1.95 |  | **1.1E-3** |  | 0.017 |  | 0.237 |  | 0.229 | 0.156 |
| Fasting plasma proinsulin | 10,026/73 |  | 1.28 |  | 1.04 |  | 1.58 |  | 0.019 |  | 0.047 |  | 0.444 |  | 0.511 | 0.401 |
| OGTT 30 min plasma proinsulin | 9270/63 |  | 1.61 |  | 1.26 |  | 2.06 |  | **1.4E-4** |  | **1.3E-4** |  | 5.8E-3 |  | 8.8E-3 | 6.6E-3 |
| OGTT 120 min plasma proinsulin | 9300/63 |  | 1.60 |  | 1.25 |  | 2.06 |  | **2.4E-4** |  | 2.8E-3 |  | 0.050 |  | 0.058 | 0.036 |
| Serum C-peptide | 9988/73 |  | 1.44 |  | 1.14 |  | 1.82 |  | **2.3E-3** |  | 0.013 |  | 0.173 |  | 0.239 | 0.229 |
| Matsuda index | 9248/63 |  | 0.63 |  | 0.50 |  | 0.80 |  | **9.8E-5** |  | **4.4E-4** |  | 0.045 |  | 0.058 | 0.022 |

CRP, C-reactive protein; HbA1c, haemoglobin A1c; OGTT, oral glucose tolerance test

Analysis includes 10,101 participants without incident AS at baseline, of whom 73 participants developed incident AS during the follow-up period. Hazard ratios (HR) are standardized. P < 2.3 x 10^-3^ (0.05/22) is considered statistically significant (bold). P<0.05 is considered as nominally significant (underlined).

P unadjusted. P* adjusted for age. P** adjusted for age and BMI. P*** Adjusted for age, BMI, smoking status and reimbursement for hypertension. P**** Adjusted for age, BMI, smoking status, reimbursement for hypertension and diabetes.

**Supplementary table S8** Principal component analysis of baseline variables, which were associated with incident AS in unadjusted Cox regression analyses, and unadjusted Cox regression analysis of the principal components associated with incident AS in the METSIM cohort. Participants with diabetes (n=1423) or AS (n=53) at baseline are excluded.

| Component |  | Factor loading | Percent variance explained | HR (95 % Cl) | *P* | |  |  |
| --- | --- | --- | --- | --- | --- | --- | --- | --- |
|  |  |  |  |  |  |  |  |  |
| PC 1 | Fasting plasma insulin  Matsuda index  C-peptide  Waist/hip ratio  Body fat mass percentage  GHbA1C | **0.949**  **-0.948**  **0.913**  **0.770**  **0.609**  **0.451** | 41.7 | 1.44 (1.15-1.80) | | **1.5E-3** |  |  |
|  |  |  |  |  |  |  |  |  |
|  |  |  |  |  |  |  |  |  |
|  |  |  |  |  |  |  |  |  |
|  |  |  |  |  |  |  |  |  |
|  |  |  |  |  |  |  |  |  |
| PC 2 | Age  Systolic blood pressure | **0.879**  **0.630** | 13.4 | 1.70 (1.40-2.08) | | **1.3E-7** |  |  |
|  |  |  |  |  |  |  |  |  |
|  |  |  |  |  |  |  |  |  |
|  |  |  |  |  |  |  |  |  |
|  | High sensitive CRP  Urine albumin excretion rate | **0.783**  **-0.509** | 11.3 | 1.10 (0.87-1.38) | | 0.438 |  |  |
| PC 3 |  |  |  |  |  |  |  |  |
|  |  |  |  |  |  |  |  |  |

CRP, C-reactive protein; HbA1c, haemoglobin A1c

CI, confidence interval; CRP, C-reactive protein; HbA1c, haemoglobin A1c

Analysis includes 8710 participants without AS at baseline, of whom 86 participants developed incident AS during the 10.8 yeas follow-up period. All variables except for age were log-transformed for statistical analysis. Bold indicates variables with significant (>0.400) loadings. P-value < 1.7 x 10^-3^ (0.05/3) is considered statistically significant (bold) and P<0.05 is considered nominally significant (underlined).

**Supplementary table S9** Principal component analysis of baseline variables, which were associated with incident AS in unadjusted Cox regression analyses, and unadjusted Cox regression analysis of the principal components associated with incident AS in the METSIM cohort. Participants with AS at baseline (n=53) and AS patients with bicuspid aortic valve (n=22) or undefined number of cusps in aortic valve (n=21) are excluded.

| Component |  | Factor loading | Percent variance explained | HR (95 % Cl) | *P* |  |  |  |
| --- | --- | --- | --- | --- | --- | --- | --- | --- |
|  |  |  |  |  |  |  |  |  |
| PC 1 | Fasting plasma insulin | **0.952** | 43.1 | 1.57 (1.22-2.03) | **4.7E-4** |  |  |  |
|  | Matsuda index | **-0.938** |  |  |  |  |  |  |
|  | C-peptide | **0.900** |  |  |  |  |  |  |
|  | Waist/hip ratio | **0.761** |  |  |  |  |  |  |
|  | GHbA1c | **0.647** |  |  |  |  |  |  |
|  |  |  |  |  |  |  |  |  |
| PC 2 | Age | **0.904** | 14.6 | 2.04 (1.61-2.59) | **2.9E-9** |  |  |  |
|  | Body fat mass percentage | **0.624** |  |  |  |  |  |  |
|  | Systolic blood pressure | **0.563** |  |  |  |  |  |  |
|  |  |  |  |  |  |  |  |  |
|  |  |  |  |  |  |  |  |  |
| PC 3 | Urine albumin excretion rate | **-0.761** | 11.5 | 0.97 (0.72-1.29) | 0.823 |  |  |  |
|  | High sensitive CRP | **0.704** |  |  |  |  |  |  |

CI, confidence interval; CRP, C-reactive protein; HbA1c, haemoglobin A1c

Analysis includes 10,101 participants without AS at baseline, of whom 73 participants developed incident AS during the 10.8 yeas follow-up period. All variables except for age were log-transformed for statistical analysis. Bold indicates variables with significant (>0.400) loadings. P-value < 1.7 x 10^-3^ (0.05/3) is considered statistically significant (bold) and P<0.05 is considered nominally significant (underlined).
